# Long Non-coding RNA EBLN3P Regulates UHMK1 Expression by Sponging miR-323a-3p and Promotes Colorectal Cancer Progression

**DOI:** 10.3389/fmed.2021.651600

**Published:** 2021-05-24

**Authors:** Xiang-hao Xu, Wen Song, Jun-hua Li, Ze-qi Huang, Ya-fang Liu, Qiang Bao, Zhi-wen Shen

**Affiliations:** ^1^Guangdong Provincial Key Laboratory of Malignant Tumor Epigenetics and Gene Regulation, Sun Yat-sen Memorial Hospital, Sun Yat-sen University, Guangzhou, China; ^2^Breast Tumor Center, Sun Yat-sen Memorial Hospital, Sun Yat-sen University, Guangzhou, China; ^3^Department of Anesthesiology, Sun Yat-sen Memorial Hospital, Sun Yat-sen University, Guangzhou, China; ^4^Department of Intensive Care Unit, Sun Yat-sen Memorial Hospital, Sun Yat-sen University, Guangzhou, China

**Keywords:** LncRNA EBLN3P, MiR-323a-3p, UHMK1, biomarker, colorectal carcinoma, metastasis

## Abstract

**Background:** Growing studies have demonstrated that long non-coding RNA (lncRNA) can act as crucial roles during the progression of various tumors, including colorectal carcinoma (CRC). We aimed to determine lncRNA endogenous bornavirus-like nucleoprotein (EBLN3P) expression in CRC and examine its influence on tumor behaviors of CRC cells.

**Materials and Methods:** Quantitative real-time polymerase chain reaction was used to determine the expression levels of EBLN3P and miR-323a-3p in CRC tissues and cell lines. Cell viability, migration, invasion, and apoptosis were assessed by Cell Counting Kit 8, colony formation, Transwell assay, wound healing assays, and flow cytometry. Bioinformatics and dual-luciferase assays were used to investigate the interaction between EBLN3P and miR-323a-3p, as well as between miR-323a-3p and U2AF homology motif kinase 1 (UHMK1). Western blot was applied for detecting the expressions of the related proteins.

**Results:** EBLN3P was highly expressed in CRC, and its high expression was distinctly associated with increased tumor size, histology/differentiation and advanced TNM stage, and poor clinical outcome of CRC patients. EBLN3P silencing significantly inhibited the proliferation and metastasis and induced the apoptosis of CRC cells. Mechanistically, overexpression of EBLN3P exhibited tumorigenic effects through downregulating the inhibitory effects of miR-323a-3p on UHMK1 expression. The correlation analysis confirmed the positive or negative association among EBLN3P, miR-323a-3p, and UHMK1.

**Conclusion:** EBLN3P promoted the development of CRC *via* targeting miR-323a-3p/UHMK1, which provided a new idea for treating CRC.

## Introduction

Colorectal carcinoma (CRC) refers to the third most prevalent carcinoma carried by males, as well as a major cause of cancer death rate worldwide, particularly in developed countries (1). A more serious scene exhibits in China, where the mortality and incidence for CRC rank second among all types of tumors (2). Despite the distinct advancements with efforts for many years in diagnosis methods and combined treatments including surgical resection and adjuvant therapies, CRC cases' 5-year surviving ratio remains at approximately 15% (3, 4). Survival probability can be distinctly improved by the use of the early detection (5). Thus, an in-depth insight into the molecularly related systems of CRC progression is urgently needed to facilitate the prevention and treatments of CRC cases.

Long non-coding RNAs (lncRNAs, with the length of >200 nucleotides) refer to one new non-coding RNA molecule family exhibiting restricted or no ability to code protein (6). There is growing evidence that lncRNAs may control epigenetic modification and chromatin remodeling to be involved in a large range of cellular processes, including growth, migratory ability, tumor differentiation, and stem cell pluripotency (7). More and more researches have revealed that lncRNAs may be novel tumor markers and therapeutic targets and exhibit antioncogenic or tumor-promotive effects in many human tumors, including CRC (8, 9). Hence, it is critical to better understand the CRC progression to explore novel tumor-related lncRNAs and to investigate their biological effects.

U2AF homology motif kinase 1 (UHMK1) is a ubiquitously expressed nuclear serine (Ser, S)/threonine (Thr, T) kinase (10). Although the biological function of UHMK1 remains largely unclear, its dysregulated expression in tumor specimens and potential regulatory effects on tumor progression have been frequently reported (11, 12). In CRC, UHMK1 overexpression and its oncogenic roles have been demonstrated (13). However, the potential mechanisms involved in its dysregulation were rarely reported.

LncRNA endogenous bornavirus-like nucleoprotein (EBLN3P) was a recently identified lncRNA, suggested to impact liver carcinoma's progressing state (14). Nevertheless, its expressing state and function in other tumors have not been investigated. In this study, we first reported its increased levels in CRC cases and further explored its tumor-related function *via in vitro* assays.

## Materials and Methods

### Tissue Collection

Ninety-five paired CRC tissue samples in this study received the collection from July 2014 to June 2016 in Sun Yat-sen Memorial Hospital, Sun Yat-sen University. No locally or systemically related treating processes were conducted in 95 cases prior to the surgery. Two experienced pathologists demonstrated all tumor and non-tumor specimens carefully. All samples received the snap-freezing and storing processes inside liquid nitrogen when resected for subsequent reverse transcriptase–polymerase chain reaction (RT-PCR) assays. Clinical and pathological characteristics were also collected for each patient. This study gained the approval from the ethics committee of Sun Yat-sen Memorial Hospital, Sun Yat-sen University. All cases included in this study provided informed consent in a written form.

### Bioinformatics Analysis

The binding sites among EBLN3P, miR-323a-3p, and UHMK1 received the prediction on starBase (http://starbase.sysu.edu.cn/index.php). “GEPIA” analyzed UHMK1 expression and its clinical significance (http://gepia.cancer-pku.cn/). *Potential miRNA binding to EBLN3P*. miRNA–EBLN3P interaction was predicted by miRanda (http://www.miranda.org/), RegRNA2(http://regrna2.mbc.nctu.edu.tw/), and starBase 2.0 (http://starbase.sysu.edu.cn/).

### Cell Lines and Cell Transfection

The Chinese Academy of Sciences (Shanghai, China) provided colonic epithelial cell line NCM460 of humans and CRC cell lines HCT116, SW480, LoVo, and HT29 cells. The culture medium for all cells was 90% Roswell Park Memorial Institute (RPMI) 1640. Cells were maintained in 5% CO_2_ and at 37°C.

Short hairpin RNA (shRNA) sequences targeting EBLN3P were designed by Yiyan Technology (Shenzhen, Guangdong, China). The shRNAs were inserted into lentiviral pHBLV/U6-Scramble-Luc-Puro^01^ vector (GenePharma, Pudong, Shanghai, China), named sh-1 and sh-2, and negative control was named sh-NC. MiR-323a-3p inhibitors were synthesized by Weizhen Biology (Jinan, Shandong, China). miR-323a-3p mimic was synthesized by Ribo Co., Ltd. (Guangdong, China). Plasmid transfections were performed with Lipofectamine 2000 (Invitrogen, Hangzhou, Zhejiang, China) in accordance with the producer's protocol. The efficiency of the transfecting process received the detection based on RT–quantitative PCR (qPCR).

### RNA Extracting Process and RT-qPCR

Overall RNA received the extraction according to tumor samples and cells with the use of Trizol reagent (Invitrogen, Nanjing, Jiangsu, China), based on the manufacturer's instructions. After purification, cDNA was synthesized from 10 μg total RNAs applying the Prime Script RT Master Mix (Takara, Nanjing, Jiangsu, China). This study conducted PCR in a real time based on the standard SYBR-Green PCR kit protocol on ABI 7600 (Applied Biosystems, Shenzhen, Guangdong, China). The relatively expressing state pertaining to miRNAs or EBLN3P received the calculation with the 2^−ΔΔ*Ct*^ methods and normalized to U6 and GAPDH, separately. Table 1 lists the primer sequences employed in this study. The qRT-PCR reactions were performed in triplicate.

**Table 1:** The primers used in this study for RT-PCR.

**Names**	**Sequences (5^**′**^-3^**′**^)**
EBLN3P: F	GTGTTGTCCCGGAAGTGCCTTCTC
EBLN3P: R	TTGAAGGTTTGCCTTCTCTGAATAG
miR-323a-3p: F	TCTAGAGGTGGTCCGTGGC
miR-323a-3p: R	CTCAACTGGTGTCGTGGA
UHMK1: F	ACGCTGTCTGTTGCTTGAACT
UHMK1: R	GGCACAATGCTGTATCATCCAC
GAPDH: F	GGAGCGAGATCCCTCCAAAAT
GAPDH: R	GGCTGTTGTCATACTTCTCATGG
U6: F	ATTGGAACGATACAGAGAAGATT
U6: R	GGAACGCTTCACGAATTTG

### Cell Proliferation Assays

Cell proliferating process received the measurement based on Cell Counting Kit 8 (CCK-8, Dojindo, Haidian, Beijing, China) by complying with the producer's instructions. Briefly, a total of 3.0 × 10^3^ cells received the seeding process inside 96-well plates' respective well. The kit was used to measure cell viability at 24, 48, and 72 h when the cells were seeded. The absorbance at 450 nm at each time point was recorded, followed by plotting of the cell proliferation curves.

### Colony Formation Assay

Cells receiving the plating process on six-well plates at 1 × 10^3^ cells per underwent the 10-day culturing process. Next, colonies received the 10-min fixing process by using 10% formaldehyde, as well as the 10-min staining process by using 1.0% crystal violet.

### Flow Cytometry

A double-staining approach based on the Vybrant Apoptosis Teat Kit#2 (Puluosai Biology, Haidian, Beijing, China) was applied to measure apoptosis. When the infecting stage was achieved, cells received the processing based on the technical manual. Five hundred microliters of 1 × annexin-binding buffer (Puluosai Biology, Haidian, Beijing, China) received the addition, and the cells that received the staining process underwent analysis based on FACSCalibur flow cytometer (no. 342973, BD Biosciences, Pudong, Shanghai) by the use of CellQuest software.

### Subcellular Fractionation

The subcellular-related fractionating process for EBLN3P received the measuring process based on PARIS Tool (Life Technologies, Haidian, Beijing, China) based on technical manual, including nuclear and cytoplasmic fraction.

### Wound Healing Assay

CRC cells (4 × 10^5^ cells/well) received the seeding process inside six-well plates and the culturing process until confluence was reached. This study used a 20-μL pipette tip for drawing one scratch in the cell monolayer. Plates received the washing process one time by using fresh medium when they have achieved the 48-h culturing process for removing non-adherent cells. When the washing process was achieved, the images of the plates were captured.

### Transwell Invasion Assays

The cell invasion was carried out by the use of one 24-well Transwell chamber (Corning, Hangzhou, Zhejiang, China). Cells received the plating process in the upper chamber under the precoating process with 2% Matrigel (BD Biosciences, Hangzhou, Zhejiang, China). The lower chambers received the loading process by using 500 μl Dulbecco modified eagle medium supplemented by 20% fetal bovine serum. One hundred percent methanol was used to fix the invaded cells, which further received the 20-min staining process with 0.1% crystal violet. A microscope was used to count the number of invaded cells.

### Animal Study

BALB/c female nude mice from Shanghai GemPharmatech (Jiangbei, Nanjing, China) were applied for *in vivo* experiments. SW480 cells stably transfected with sh-NC and sh-EBLN3P-1 at a density of 4 × 10^6^ were injected into nude mice (*n* = 6). Every 4 days, the tumor volumes were recorded. A formula (length × width^2^ × 0.5) was used to calculate the volumes. Twenty-eight days later, tumors were excised from killed mice and weighed for further analysis. The animal-related protocol was approved by the Animal Research Ethics Committee of Sun Yat-sen Memorial Hospital, Sun Yat-sen University.

### Luciferase Reporter Assay

EBLN3P fragment was supplemented by the assessed miR-323a-3p binding site; the binding site's wide-type or mutant putative sequences received the subcloning process in one pmirGLO dual-luciferase vector (Bafeier Biology, Shijingshan, Beijing, China) for forming the pmiRGLO-EBLN3P mutant (EBLN3P-mut) or reporter vector pmiRGLO- EBLN3P wild type (EBLN3P-wt). EBLN3P-wt or EBLN3P-mut received the cotransfecting process by using negative control (miRNC) or miR-323a-3p mimics inside SW480 and HCT116 cells based on Lipofectamine 2000. Similarly, the reporter vector pmiRGLO-UHMK1–wild type (UHMK1-wt) or pmiRGLO-UHMK1–mutant (UHMK1-mut) received the synthesizing process. At 48 h when the transfecting process was achieved, the corresponding activities of luciferase received the measuring process.

### RNA Pull-Down Assays

RNA pull-down assays were carried out by complying with RNA Pull-Down Tool's guidelines (Pierce, Shenzhen, Guangdong, China). In brief, EBLN3P and EBLN3P antisense (negative control) received the cloning process inside pcDNA 3.1 vector, followed by transcription by the use of T7 RNA polymerase (Promega, Pudong, Shanghai, China). The mentioned RNAs received the labeling process based on biotin. After washing, this study carried out RT-PCR for measuring the coprecipitated RNAs.

### Western Blot Analysis

Cells were lysed with RIPA lysis buffer (E-BC-R327, Elabscinece, Pudong, Shanghai, China) for total protein extraction. Equal amounts of proteins (50 μg) were separated by 10% sodium dodecyl sulfate–polyacrylamide gel electrophoresis (Yita Technology, Haidian, Beijing, China) and transferred onto a polyvinylidene fluoride membrane (Biomart, Haidian, Beijing, China). The membrane was incubated with 5% non-fat milk for 1 h at room temperature, followed by incubation with corresponding primary antibodies against cleaved caspase-3, caspase-3, cleaved caspase-9, caspase-9, N-cadherin, vimentin, E-cadherin, or GAPDH overnight at 4°C. The membrane was then washed three times with phosphate-buffered saline, followed by incubation with horseradish peroxidase–conjugated secondary antibody at room temperature for 1 h. All antibodies were purchased from Aviva Biology (Daxing, Beijing, China). An enhanced chemiluminescence kit (Abcam) was used to detect the Western blot bands.

### Statistical Analysis

Based on SPSS 18.0 (SPSS Inc., Chicago, IL, USA), the authors conducted the statistics-related analyzing processes. By performing individual two-tailed Student *t* testing process, the data received the analysis. This study adopted receiver operating characteristic (ROC) curve for analyzing the efficacy of EBLN3P in the diagnosis of CRC specimens from non-tumor specimens. This study conducted the χ^2^ testing process for assessing the relationship of EBLN3P expressing states with clinicopathologically related characteristics. The authors obtained surviving curves of disease-free survival (DFS) and overall survival (OS) using Kaplan–Meier estimates. In addition, the authors determined the differences between groups based on the log-rank testing process. Prognostic significance pertaining to each variable received the analysis using the Cox regression model. *p* < 0.05 exhibited statistical significance.

## Results

### EBLN3P Displayed a Significant Up-Regulation in CRC Cases

**Figure 1:**
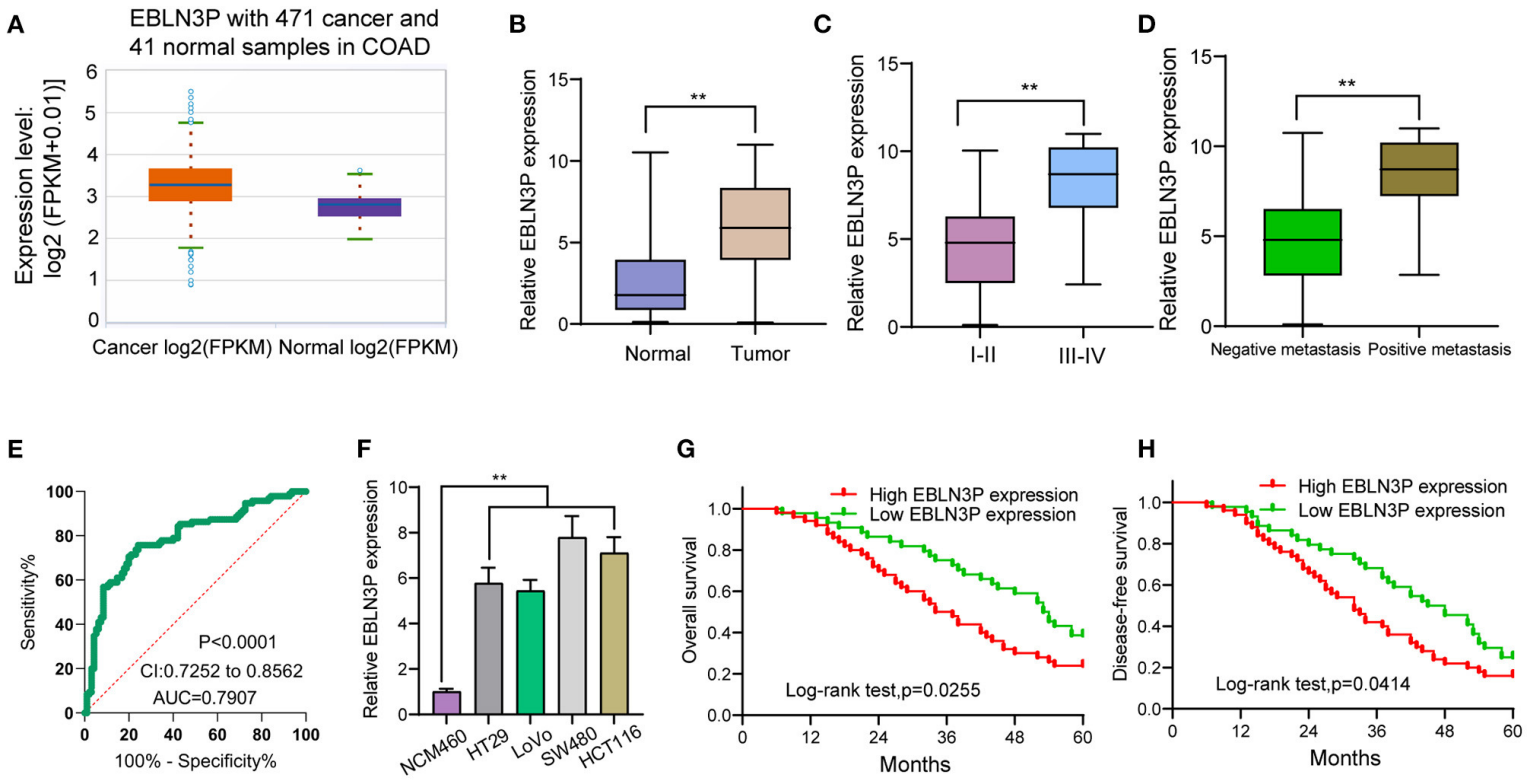
Increased expression of EBLN3P in CRC and its clinical significance. **(A)** The expression of EBLN3P in 471 CRC specimens and 41 non-tumor specimens from TCGA datasets. **(B)** The levels of EBLN3P in 95 pairs of primary CRC tissues and the matched non-tumor specimens by RT-PCR. **(C,D)** Higher levels of EBLN3P were observed in specimens with advanced stages and positive metastasis than those with early stages and negative metastasis. **(E)** ROC assays for the diagnostic value of EBLN3P expression in CRC specimens. **(F)** RT-PCR for the levels of EBLN3P in four CRC cell lines (HT29, LoVo, SW480, and HCT116) and human colonic epithelial cell lines NCM46 cells. **(G,H)** The survival assays of 95 CRC patients according to the mean expression of EBLN3P in 95 CRC specimens. **p < 0.01.

First, we performed RT-PCR to examine the expression of EBLN3P in 95 CRC patients. According to the results, EBLN3P expression was distinctly increased in CRC specimens compared with matched non-tumor tissues (Figure 1A). We also observed higher levels of EBLN3P in CRC specimens with advanced stages and positive metastasis than those with early stages and negative metastasis (Figures 1B,C). Using TCGA dataset, we also observed an increased expression of EBLN3P in CRC samples (Figure 1D). According to the ROC assays, our group observed that high EBLN3P expression had 0.7907 AUC [95% confidence interval (CI) = 0.7252–0.8562] in terms of CRC (Figure 1E). Then, the level of EBLN3P was checked in CRC cell lines and NCM460. In Figure 1F, EBLN3P overexpressed in four cell lines compared with NCM460. Overall, the research presented here suggested EBLN3P as a novel modulator in CRC progression.

### High-Expression Level of EBLN3P Predicts Weak Prognostic Process Inside CRC Cases

To explore the clinical significance of EBLN3P expression in CRC patients, 95 patient samples were divided into low group (<5.83) and high groups (>5.83) according to the mean expression of EBLN3P (5.83). As shown in Table 2, we observed that high EBLN3P expression was associated with tumor size (*p* = 0.032), histology/differentiation (*p* = 0.013), TNM stage (*p* = 0.010), and distant metastasis (*p* = 0.029). To further study the clinical value of EBLN3P in CRC cases, we analyzed survival data of all 95 CRC cases using a Kaplan–Meier survival analysis. We observed that the OS (Figure 1G, *p* = 0.0255) and DFS (Figure 1H, *p* = 0.0414) of cases with high EBLN3P expression were distinctly shorter than those of cases with low BLN3P expression. More importantly, in a multivariate Cox model, we found that EBLN3P expression was an independent poor prognostic factor for both OS [hazard ratio (HR) = 2.863, 95% CI = 1.372–4.675, *p* = 0.008; Table 2] and DFS (HR = 3.152, 95% CI = 1.347–5.183, *p* = 0.004; Table 3) in CRC.

**Table 2:** Correlation between EBLN3P expression and clinicopathological features of CRC.

**Clinicopathological features**	**No. of cases**	**EBLN3P expression**		***P*-value**
		**High**	**Low**	
Age				0.931
≤60 years	49	26	23	
>60 years	46	24	22	
Gender				0.706
Male	51	30	31	
Female	44	20	24	
Tumor size				0.032
≤5 cm	46	19	27	
>5 cm	49	31	18	
Histology/differentiation				0.013
Well + moderate	55	23	32	
Poor	40	27	13	
TNM stage				0.010
I–II	59	25	34	
III–IV	36	25	11	
Distant metastasis				0.029
Yes	68	31	37	
No	27	19	8	

**Table 3:** Multivariate analyses for disease-free survival and overall survival by Cox regression model.

**Variable**	**Overall survival**			**Disease-free survival**		
	**HR**	**95% CI**	***P***	**HR**	**95% CI**	***P***
Age	0.893	0.456–1.783	0.231	1.132	0.567–2.034	0.134
Gender	1.231	0.567–2.055	0.346	1.445	0.756–2.214	0.214
Tumor size	1.542	0.783–2.341	0.087	1.491	0.858–2.441	0.094
Histology/differentiation	2.984	1.238–4.868	0.015	3.018	1.472–5.229	0.008
TNM stage	3.258	1.458–5.672	0.001	3.587	1.558–6.582	0.001
Distant metastasis	3.135	1.243–5.252	0.011	3.324	1.343–5.677	0.007
EBLN3P expression	2.863	1.372–4.675	0.008	3.152	1.347–5.183	0.004

### EBLN3P Knockdown Suppressed the Proliferation of CRC Cell Lines

**Figure 2:**
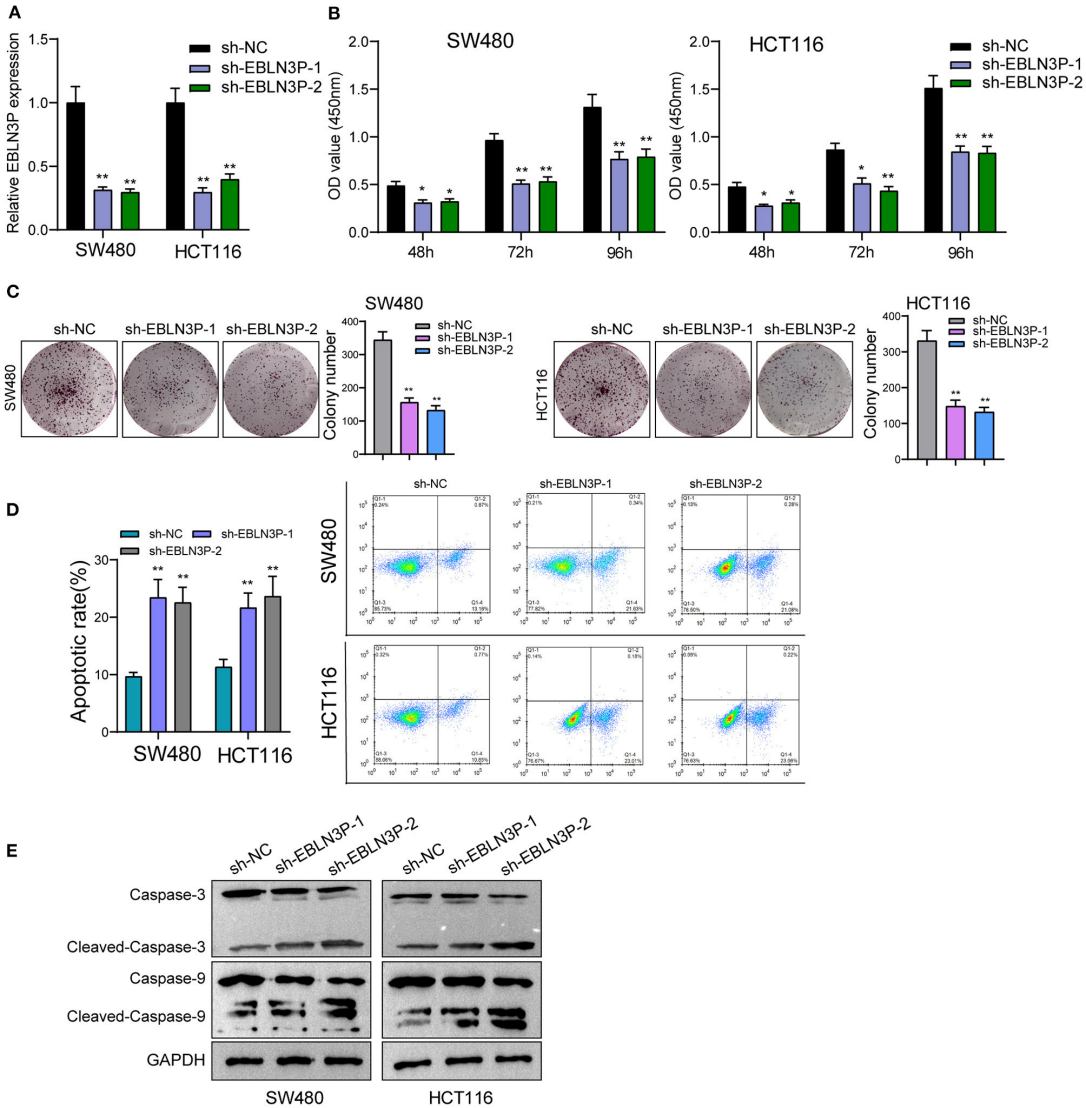
Knockdown of EBLN3P suppressed the proliferation of SW480 and HCT116 cells. **(A)** RT-PCR for the expression of EBLN3P in SW480 and HCT116 cells transfected with sh-EBLN3P-1, EBLN3P-2, or sh-NC. **(B)** CCK-8 assays determined the cell ability in SW480 and HCT116 cells transfected with sh-EBLN3P-1, EBLN3P-2 or sh-NC. **(C)** Clone formation capacity of SW480 and HCT116 cells was assessed by the clone formation assays. **(D)** Apoptotic rate of CRC cells was shown after the transfection. **(E)** Western blot for the expression of cleaved caspase-3, caspase-3, cleaved caspase-9, and caspase-9 in SW480 and HCT116 cells transfected with sh-EBLN3P-1, EBLN3P-2, or sh-NC. *p < 0.05; **p < 0.01.

**Figure 3:**
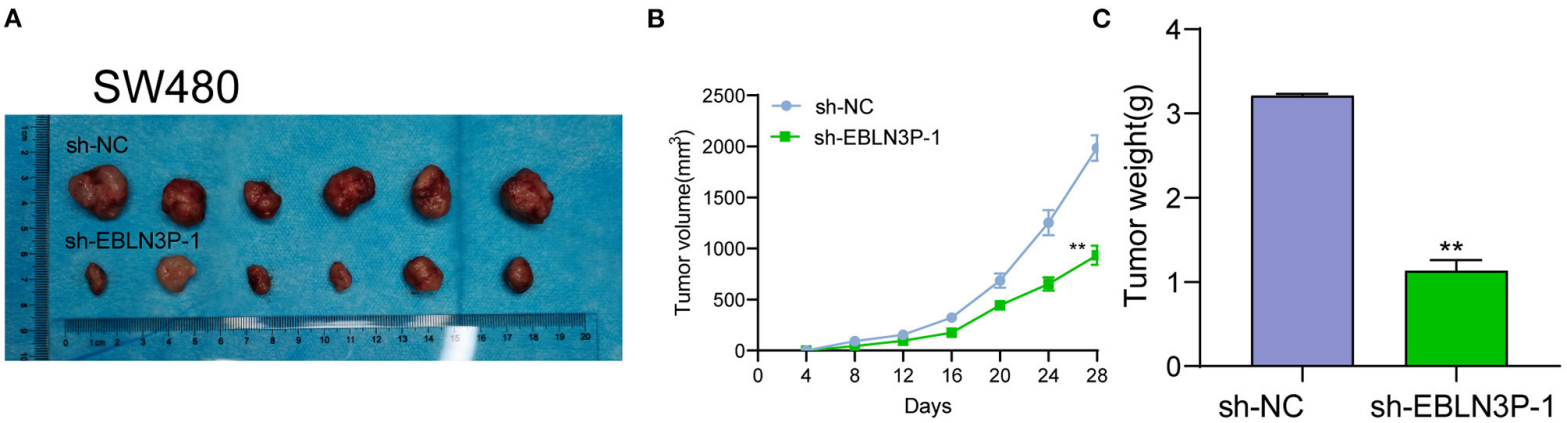
EBLN3P promotes CRC tumor growth *in vivo*. **(A)** Representative images of the subcutaneous tumors formed by EBLN3P-knockdown and control SW480 cells. **(B)** Tumor volumes were detected every 7 days. **(C)** The subcutaneous tumor weights were detected at the 28th day after injection. **p < 0.01.

The distinct overexpression of EBLN3P in CRC prompted us to evaluate the biological roles of EBLN3P in CRC cells. The specific lentiviral vector expressing EBLN3P shRNAs was transected to SW480 and HCT116 to decrease the expression of EBLN3P. A distinctly decreased expression of EBLN3P in SW480 and HCT116 cells transfected with sh-1 and sh-2 was demonstrated using RT-PCR (Figure 2A). CCK-8 experiments revealed that the proliferation rate of SW480 and HCT116 cells transfected with sh-1 or sh-2 was significantly decreased compared with negative control (sh-NC) (Figure 2B). Colony formation assays indicated that the cells transfected with sh-1 or sh-2 exhibited a weakened capacity for colony formation compared with the control group in SW480 and HCT116 cells (Figure 2C). Flow cytometry indicated that knockdown of EBLN3P promoted apoptosis of SW480 and HCT116 cells (Figure 2D). Moreover, we examined the influence of EBLN3P on the expression of caspase-3 and caspase-9, and the results of Western blot revealed that knockdown of EBLN3P distinctly resulted in the suppression of the expression of caspase-3 and caspase-9, while it promoted the expression of cleaved caspase-3 and cleaved caspase-9 (Figure 2E). On the other hand, we also performed *in vivo* assays to explore the influence of EBLN3P knockdown on tumor growth. As shown in Figure 3A, we observed that the tumor growth speed was slower on nude mice after subcutaneous injection with sh-EBLN3P-1 than control group (Figure 3A). Besides, we found that the tumor volume and weight were apparently lessened in sh-EBLN3P-1 group compared with control group (Figures 3B,C).

### EBLN3P Knockdown Suppressed the Metastasis of CRC Cell Lines

**Figure 4:**
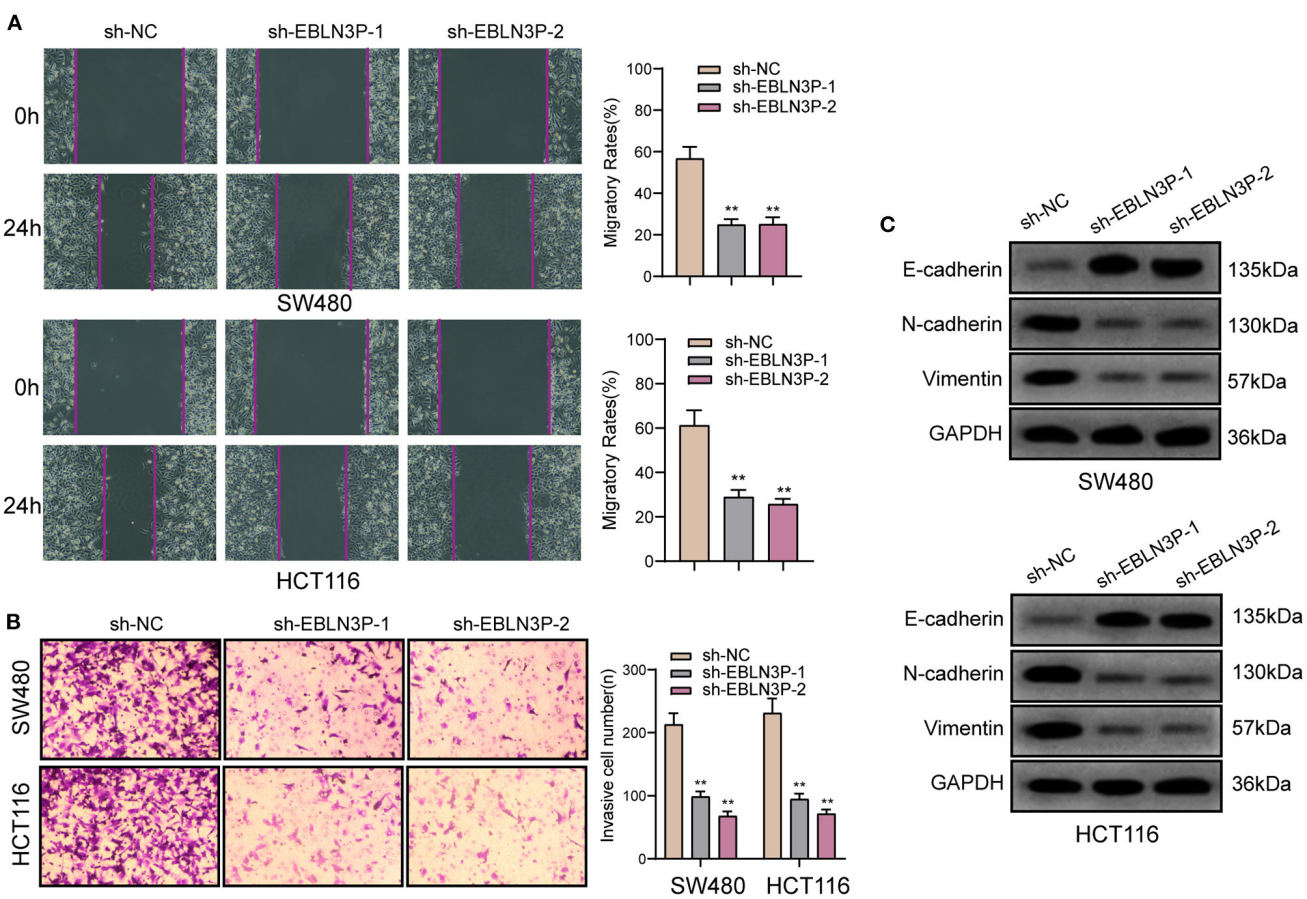
The antioncogenic roles of EBLN3P knockdown on the migration of invasion of SW480 and HCT116 cells. **(A)** Scratch assay following EBLN3P knockdown. **(B)** Transwell assays following EBLN3P knockdown. **(C)** Western blot assays for the expression of EMT markers. **p < 0.01.

To further explore whether EBLN3P has a functional effect on tumor metastasis ability, we performed a scratch-wound healing assay and observed that compared with SW480 and HCT116 cells transfected with sh-NC, those transfected with sh-1 or sh-2 showed a significantly increased migration 24 h after transfection (Figure 4A). In addition, Transwell assays suggested that EBLN3P knockdown notably enhanced the invasion of SW480 and HCT116 cells (Figure 4B). It has been demonstrated that epithelial–mesenchymal transition (EMT) is one of the important indicators for tumor metastasis (15). For the detection of EMT-related proteins, Western blot was performed. As shown in Figure 4C, we observed that EBLN3P knockdown induced a pronounced decrease in N-cadherin and vimentin expression and an increase in E-cadherin expression. Collectively, we concluded that EBLN3P was involved in CRC metastasis, and functioned as an oncogenic lncRNA.

### EBLN3P Acted as miR-323a-3p Sponge in CRC Cells

**Figure 5:**
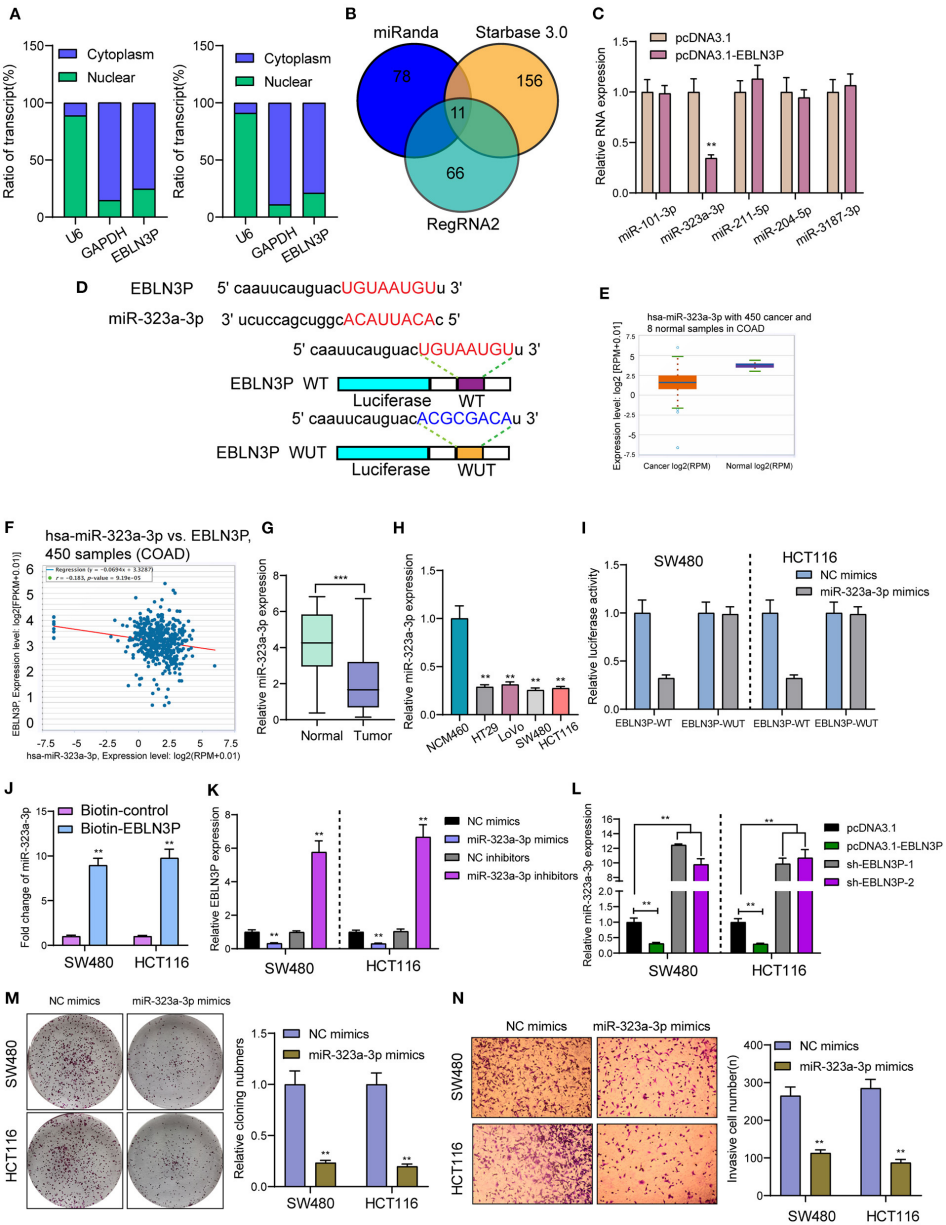
EBLN3P acts as a sponge for miR-323-3p in the cytoplasm. **(A)** Relative EBLN3P expression levels in nuclear and cytosolic fractions of SW480 and HCT116 cells. **(B)** Potential miRNA binding to EBLN3P. miRNA–EBLN3P interaction was predicted by miRanda, RegRNA2 and starBase 2.0, of which 11 were overlapped. **(C)** RT-PCR confirmed miR-323-3p expression was decreased in SW480 cells after overexpression of EBLN3P. **(D)** Schematic outline of the predicted binding sites for miR-323-3p on EBLN3P and the mutation pattern of EBLN3P. **(E)** The expression of miR-323-3p in 450 CRC specimens and 8 non-tumor tissues based on TCGA datasets. **(F)** The correlation between EBLN3P and miR-323-3p expression analyzed in 450 CRC samples. **(G)** Increased expression of miR-323-3p in normal specimens compared to matched CRC specimens from our cohort. **(H)** RT-PCR for the levels of miR-323-3p in four CRC cell lines (HT29, LoVo, SW480, and HCT116) and human colonic epithelial cell lines NCM46 cells. **(I)** Luciferase activity assay revealed that miR-323-3p significantly decreased the luciferase detection of EBLN3P-Wt, but not EBLN3P-mut in SW480 and HCT116 cells. **(J)** RNA pull-down verifying miR-323-3p binding to EBLN3P. **(K)** The expression of EBLN3P in SW480 and HCT116 cells after overexpression or knockdown of miR-323-3p. **(L)** The expression of miR-323-3p in SW480 and HCT116 cells after overexpression or knockdown of EBLN3P. **(M,N)** Colony formation assays and Transwell assays for the functional exploration of miR-323-3p. **p < 0.01, ***p < 0.01.

It has been known to us that lncRNAs exhibit their effects according to subcellular distribution (16). To determine the cellular location of EBLN3P, SW480 and HCT116 cells were isolated into nuclear and cytoplasmic fractions. Then, we observed that EBLN3P was mainly expressed in the cytoplasm (Figure 5A). To test this hypothesis, we used three bioinformatics databases (miRanda, RegRNA2, and starBase v2.0) for the prediction of the possible interaction between miRNAs and EBLN3P. Our group observed that EBLN3P contained multiple miRNA binding sites (Figure 5B). Then, we chose the top five miRNAs (miR-101-3p, miR-323-3p, miR-211-5p, miR-204-5p, and miR-3187-3p) in starBase v2.0 for further RT-PCR experiments and found that overexpression of EBLN3P suppressed the expression of miR-323a-3p, whereas the levels of miR-101-3p miR-211-5p, miR-204-5p, and miR-3187-3p remained unchanged (Figure 5C). Then, we chose miR-323a-3p for subsequent experiments. The combined sequences between EBLN3P and miR-323a-3p are shown in Figure 5D. Previously, miR-323a-3p has been demonstrated to suppress the proliferation and metastasis of CRC cells (17). Using TCGA datasets, we observed a lower level of miR-323a-3p in CRC specimens compared to normal tissues (Figure 5E). A negative association between miR-323a-3p expression and EBLN3P expression was also observed in 450 CRC samples (Figure 5F). In addition, the results of RT-PCR also showed that miR-323a-3p was lowly expressed in CRC specimens from our cases and for CRC cell lines (Figures 5G,H). Luciferase reporter assay showed that upregulation of miR-323a-3p could decrease EBLN3P-WT activity, but it had no effect on EBLN3P-MUT in both SW480 and HCT116 cells (Figure 5I). Further RNA pull-down also confirmed miR-323a-3p may target EBLN3P (Figure 5J). Moreover, overexpression of miR-323a-3p was found to suppress EBLN3P expression, whereas miR-323a-3p downregulation displayed an opposite result (Figure 5K). Similarly, the transfection of EBLN3P suppressed the expression of miR-323a-3p, whereas EBLN3P knockdown displayed an opposite result (Figure 5L). Moreover, we performed Transwell invasion assays and colony formation assays to determine the function of miR-323a-3p in CRC cells, finding that overexpression of miR-323a-3p distinctly suppressed the proliferation and invasion of SW480 and HCT116 cells (Figures 5M,N). Our findings suggest EBLN3P acted as miR-323a-3p sponge in CRC cells.

### EBLN3P Increased UHMK1 Expression *via* Sponging miR-323a-3p

**Figure 6:**
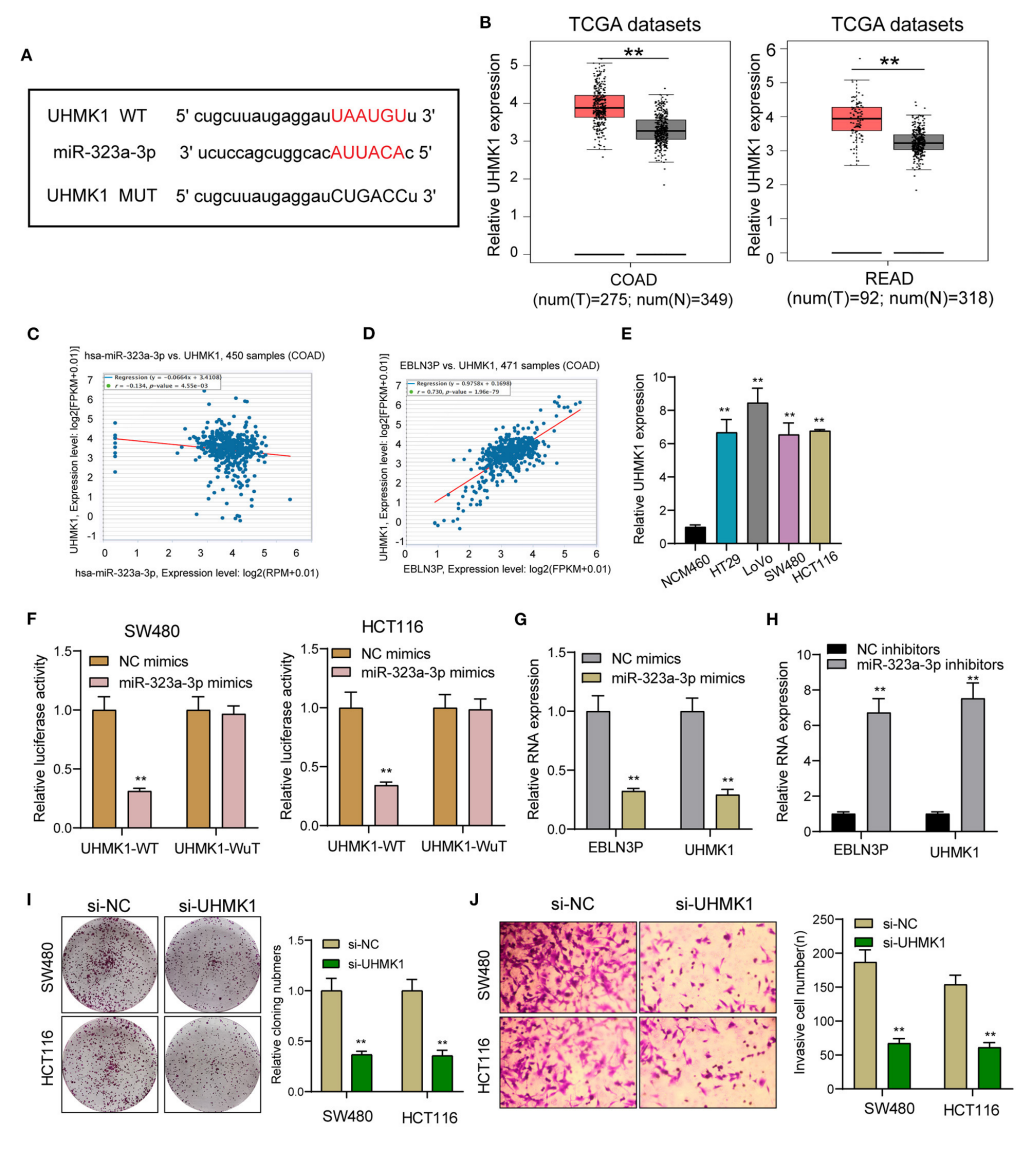
UHMK1 expression was a potential target of miR-323a-3p. **(A)** Sequence alignment of human miR-323-3p with 3′-UTR of UHMK1 mRNA predicted by TargetScan. **(B)** UHMK1 expression in 275 CRC specimens and 349 non-tumor specimens according to the data of TCGA datasets. **(C)** The correlation between UHMK1 and miR-323-3p expression analyzed in 450 CRC samples. **(D)** The correlation between UHMK1 and EBLN3P expression analyzed in 471 CRC samples. **(E)** Decreased UHMK1 mRNA expression was observed in four CRC cells compared to NCM460 cells. **(F)** The luciferase activity of the wild-type UHMK1 3′-UTR (Wt) and mutant UHMK1 3′-UTR (Mut) cotransfected with miR-323-3p mimics or NC mimics was measured. **(G)** The levels of EBLN3P and UHMK1 in CRC cells transfected with miR-323-3p mimics or NC mimics by RT-PCR. **(H)** The levels of EBLN3P and UHMK1 in CRC cells transfected with miR-323-3p mimics or NC inhibitors by RT-PCR. **(I,J)** Colony formation assays and Transwell assays for the functional exploration of UHMK1 knockdown. **p < 0.01.

**Figure 7:**
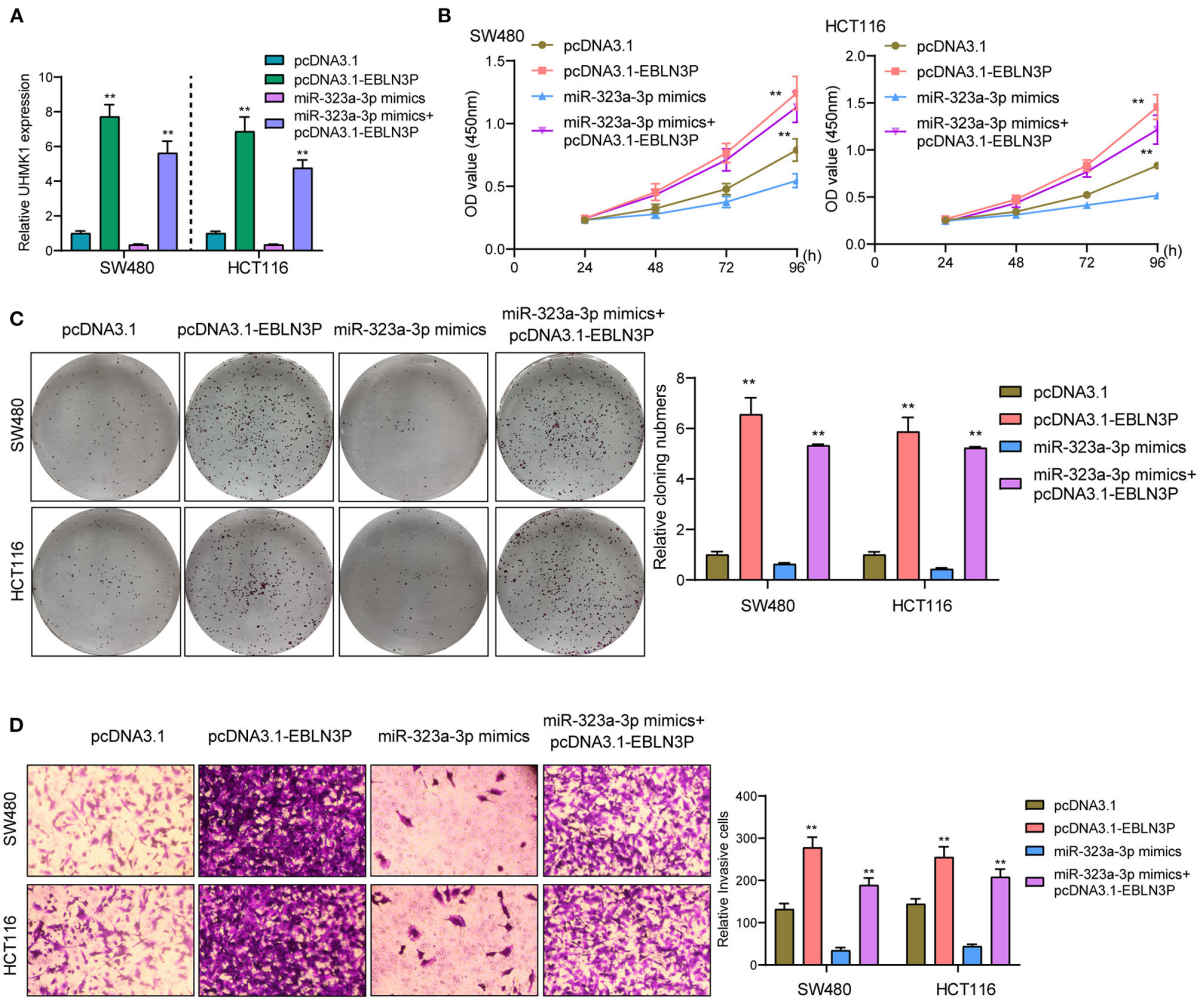
EBLN3P promoted CRC progression *via* modulating miR-323-3p/ UHMK1 axis. **(A)** RT-PCR determined the expression of UHMK1 in SW480 and HCT116 cells transfected with pcDNA3.1, pcDNA3.1-EBLN3P, miR-323-3p mimics, or miR-323-3p mimics + pcDNA3.1–EBLN3P. **(B–D)** The ability of SW480 and HCT116 cells transfected with the above factors on the proliferation and invasion was determined using CCK-8, colony formation assays, and Transwell assays. **p < 0.01.

This study employed miRanDa (www.microrna.org) and TargetScan 6.2 (www.targetscan.org) for assessing the probable miR-323a-3p target. The authors reported UHMK1–3′-UTR to cover the complementary sequence for miR-323a-3p (Figure 6A). UHMK1 expression was distinctly increased in CRC specimens in comparison with non-tumor tissues by analyzing TCGA datasets (Figure 6B). The negative association between miR-323a-3p and UHMK1 and the positive association between EBLN3P and UHMK1 was demonstrated according to the data of TCGA datasets (Figures 6C,D). We also examined the levels of mRNA UHMK1 in CRC cell lines, confirming it was overexpressed (Figure 6E). As revealed from the outcomes of the Luciferase reporter test, upregulation of miR-323a-3p could decrease UHMK1-WT activity, but it had no effect on UHMK1-MUT in both SW480 and HCT116 cells (Figure 6F). RT-PCR further suggested that miR-323a-3p overexpression resulted in the suppression of EBLN3P and UHMK1 expression, while miR-323a-3p knockdown resulted in the promotion of EBLN3P and UHMK1 expression (Figures 6G,H). We also observed that knockdown of UHMK1 suppressed the proliferation and invasion of CRC cells (Figures 6I,J). Finally, we performed rescue experiments, finding that overexpression of EBLN3P reversed the suppression of miR-323a-3p mimics on the expression of UHMK1 (Figure 7A). In addition, in a series of functional experiments, we observed that the transfection of miR-323a-3p mimics reversed the distinct suppression of the transfection of pcDNA3.1-EBLN3P on the proliferation and invasion of SW480 and HCT116 cells (Figures 7B–D). Thus, our findings suggest EBLN3P may promote CRC progression *via* regulating miR-323a-3p/UHMK1 axis.

## Discussion

In our nation, CRC's incidence and death rate merely fall behind those of lung and liver carcinoma, which rise yearly, particularly among younger generations (18). Early diagnosis is very important to improve the clinical prognosis of CRC cases. Although many methods exist for the diagnosis of CRC, the sensitive diagnostic methods are limited (19). In recent years, growing studies have revealed the potential of lncRNAs used as novel diagnostic and prognostic biomarkers for CRC cases (20, 21). In this study, we identified a novel CRC-related lncRNA, EBLN3P which was demonstrated to be overexpressed in 95 CRC cases and cell lines. We also showed its diagnostic value in screening CRC specimens from normal tissues using ROC assays. Clinical assays revealed that high EBLN3P expression was associated with tumor size, Histology/differentiation, TNM stage and poor prognosis. Our findings suggest EBLN3P as a novel biomarker for CRC cases.

In recent years, growing studies have revealed that lncRNAs serve as regulators in CRC progression (22). For instance, lncRNA SNHG7, an overexpressed factor in CRC specimens, was found to promote the proliferation and metastasis of tumor cells *via* upregulating GALNT1 (23). LncRNA SLCO4A1-AS1 was highly expressed in CRC cells, and both *in vitro* and *in vivo* assays revealed that its knockdown suppressed the growth and migration of CRC cells *via* Wnt pathway (24). LncRNA-ZFAS1, whose upregulation was induced by SP1, was shown to accelerate the proliferation and invasion of CRC cells through the miRNA-150-5p/VEGFA axis (25). The findings suggest lncRNAs as novel players in CRC progression. Previously, Hang et al. first reported that EBLN3P was overexpressed in liver cancer. Then, they performed functional assays, which revealed that forced EBLN3P expression resulted in the promotion of the proliferation and metastasis of liver cancer cells *via* alteration of miRNA-144-3p/DOCK4 signal (14). However, to our best knowledge, the expression and function of EBLN3P in other tumor types remained largely unclear. In this study, we also performed *in vitro* experiments, finding that knockdown of EBLN3P suppressed the proliferation, migration, and invasion of SW480 and HCT116 and promoted apoptosis, suggesting EBLN3P as a tumor promoter in CRC progression. Our findings were consistent with the oncogenic roles of EBLN3P in liver cancer. To explore the related mechanisms, we performed Western blot to determine the effects of EBLN3P on EMT pathways, confirming that EBLN3P knockdown suppressed the activity of EMT pathways, which could explain the reason that EBLN3P could contribute to the abilities of metastaticity.

For the in-depth exploration of the basic molecular systems allowing EBLN3P to regulate downstream effectors inside CRC, this study first reported its site in carcinoma cells, given that lncRNA functions are dependent on its subcellular localization (26). Cytosolic lncRNAs are capable of modulating mRNA stability and protein localization and act as microRNA sponge (27). In this study, we observed that EBLN3P was mainly expressed both in the cytoplasm. Bioinformatics analyses predicted miR-323a-3p targeting sites on EBLN3P. Luciferase reporter assay, binding site mutation analysis, and qPCR further verified that EBLN3P is a genuine target of miR-323a-3p. We also observed that miR-323a-3p expression was distinctly upregulated in SW480 and HCT116 cells, and the levels of EBLN3P were regulated by the transfection of miR-323a-3p mimics or miR-323a-3p inhibitors. Previously, a decreased expression of miR-323a-3p has been reported, and its overexpression suppressed the proliferation and metastasis of CRC cells, (17) which was consistent with our findings. Thus, together with previous findings, EBLN3P may display its oncogenic roles *via* sponging miR-323a-3p.

UHMK1 refers to one nuclear serine/threonine kinase exhibiting a U2AF homology motif and receiving phosphorylating process. Originally, this study found it regulating the stathmin's function (28). In recent years, UHMK1 dysregulating process or mutating process was suggested as one high-penetrant element inside various tumors of humans (e.g., gastric cancer as well as CRC) (12, 13). Here, UHMK1 exhibited a significant expressing state inside CRC. As confirmed by bioinformatics analysis and luciferase reporting element test, UHMK1 acts as miR-323a-3p's direct target. According to the correlation study, UHMK1 expressing state displayed a negative relationship to miR-323a-3p, while positively associated with EBLN3P in 471 CRC samples from TGCA datasets. Furthermore, we also found that the transfection of miR-323a-3p mimics reversed the distinct suppression of the transfection of pcDNA3.1-EBLN3P on the proliferation and invasion of SW480 and HCT116 cells. Thus, our findings suggest EBLN3P may promote CRC progression by increasing UHMK1 expression *via* sponging miR-323a-3p.

Although our study showed that EBLN3P acts as an oncogene in CRC, there are several limitations that exist in this study such as limited clinical specimens, the function of EBLN3P/miR-323a-3p/UHMK1 axis in CRC *in vivo*, and the association of EBLN3P and other potential miRNAs. Moreover, the specific role of EBLN3P-mediated regulatory mechanism in CRC needs further investigation.

## Conclusion

In summary, an EBLN3P-miR-323a-3p-UHMK1 regulating axis inside CRC pathogenic mechanism received the identification here. EBLN3P is an oncogenic lncRNA facilitating CRC tumor formation through the way of being one ceRNA regulating UHMK1's expressing state *via* sponging miR-323a-3p. Here, miR-323a-3p is proven as one probable therapeutic aim for managing CRC.

## Data Availability Statement

The data in this study did not include microarray data. The original contributions presented in the study are included in the article, further inquiries can be directed to the corresponding author/s.

## Ethics Statement

The studies involving human participants were reviewed and approved by Sun Yat-sen Memorial Hospital, Sun Yat-sen University. The patients/participants provided their written informed consent to participate in this study. The animal study was reviewed and approved by Sun Yat-sen Memorial Hospital, Sun Yat-sen University.

## Author Contributions

X-hX, QB, and Z-wS conceived and designed the experiments. X-hX, WS, and J-hL performed the experiments. Z-qH and Y-fL analyzed the data. X-hX and QB wrote the paper. All authors read and approved the final manuscript.

## Conflict of Interest

The authors declare that the research was conducted in the absence of any commercial or financial relationships that could be construed as a potential conflict of interest.
